# The PXR rs7643645 Polymorphism Is Associated with the Risk of Higher Prostate-Specific Antigen Levels in Prostate Cancer Patients

**DOI:** 10.1371/journal.pone.0099974

**Published:** 2014-06-12

**Authors:** Octavio D. Reyes-Hernández, Libia Vega, Miguel A. Jiménez-Ríos, Pedro F. Martínez-Cervera, Juan A. Lugo-García, Leticia Hernández-Cadena, Patricia Ostrosky-Wegman, Lorena Orozco, Guillermo Elizondo

**Affiliations:** 1 Laboratorio de Genética y Diagnóstico Molecular, Hospital Juárez de México, México, D.F., México; 2 Departamento de Toxicología, Centro de Investigación y Estudios Avanzados del Instituto Politécnico Nacional, México, D.F., México; 3 Departamento de Urología, Instituto Nacional de Cancerología, México, D.F., México; 4 Departamento de Salud Ambiental, Instituto Nacional de Salud Pública, México, D.F., México; 5 Instituto de Investigaciones Biomédicas, Universidad Nacional Autónoma de México, México, D.F., México; 6 Laboratorio de Inmunogenómica y Enfermedades Metabólicas, Instituto Nacional de Medicina Genómica, México, D.F., México; 7 Departamento de Biología Celular, Centro de Investigación y Estudios Avanzados del Instituto Politécnico Nacional, México, D.F., México; Southern Illinois University School of Medicine, United States of America

## Abstract

Levels of enzymes that determine testosterone catabolism such as CYP3A4 have been associated with prostate cancer (PCa) risk. Although some studies have related CYP3A4*1B allele, a gene polymorphism that modifies CYP3A4 expression level, with PCa risk, others have failed, suggesting that additional genetic variants may be involved. Expression of CYP3A4 is largely due to the activation of Pregnane X Receptor (PXR). Particularly, rs2472677 and rs7643645 *PXR* polymorphisms modify CYP3A4 expression levels. To evaluate whether *PXR-HNF3β*/T (rs2472677), *PXR-HNF4/G* (rs7643645), and CYP3A4*1B (rs2740574) polymorphisms are associated with PCa a case control-study was performed. The multiple testing analysis showed that the *PXR-HNF4/G* polymorphism was associated with higher levels of prostate-specific antigen (PSA) in patients with PCa (OR = 3.99, p = 0.03). This association was stronger in patients diagnosed at the age of 65 years or older (OR = 10.8, p = 0.006). Although the CYP3A4*1B/*1B genotype was overrepresented in PCa patients, no differences were observed in the frequency of this and *PXR-HNF3β*/T alleles between controls and cases. Moreover, no significant association was found between these polymorphisms and PSA, Gleason grade, or tumor lymph node metastasis.

## Introduction

Prostate cancer (PCa) is the second most common cancer worldwide in males and one of the most common causes of death in men (IARC, 2008). The etiology of the disease involves several factors, including ethnicity, older age, family history, and genetic and environmental factors [1], [2]. It has been established that steroid hormone levels, in particular androgens, affect the risk of developing PCa [3], [4]. Testosterone and predominantly its metabolite dihydrotestosterone interact with the androgen receptor, which leads to the expression of genes involved in the growth of the prostate and the proliferation of prostate cancer cells [5]. Several cytochromes P450s (CYPs) are involved in the synthesis and catabolism of hormones, such as testosterone [6]. The bioavailability of dihydrotestosterone is decreased by CYP3A4, which mediates the 2*β*-, 6β-, and 15β- hydroxylation of testosterone in the liver and prostate [7]–[9]. Therefore, it has been hypothesized that low levels and/or decreased CYP3A4 activity might result in a lower capacity to inactivate testosterone favoring its conversion to dihydrotestosterone and increasing the risk of developing PCa. In fact, decreased expression of CYP3A4 has been found in prostatic tissues from PCa patients compared to 93% for benign epithelium, and only 75% of prostate tumors expressed CYP3A4 [10].

Interindividual variation in CYP3A4 levels of up to 60-fold have been reported [11], [12], and it has been suggested that most of the variability can be explained by genetic factors [13]. The *CYP3A4* gene is highly polymorphic, and to date, 43 different *CYP3A4* polymorphisms have been reported (http://www.cypalleles.ki.se/), of which CYP3A4*1B is one of the most common. CYP3A4*1B is a single nucleotide polymorphism (SNP) (rs2740574) that introduces an A to G substitution at position −290, which is located in the nifenipine specific response element of the promoter of the *CYP3A4* gene. While some studies have reported associations between the CYP3A4*1B allele and higher clinical grade of PCa [14], [15], others have failed to observe an association between the presence of the CYP3A4*1B polymorphism and prostate cancer susceptibility [16], [17]. Controversial data regarding the association of CYP3A4*1B and CYP3A4 activity have also been reported [18], [19], suggesting that other genetic variants may be involved, such as transcription factors that mediate CYP3A4 expression.

Pregnane X receptor (PXR, NR1I2) is a member of the steroid nuclear receptor family of ligand activated transcription factors. Activated-PXR forms a heterodimer with 9-cis-retinoic acid X receptor (RXR) and binds to a nuclear receptor response element in the 5′-flanking region of its target genes. Induction of CYP3A4 is largely due to the activation of PXR [20]. Therefore, genetic variants of *PXR* that alter PXR protein levels or its transactivation potential may have an important impact on CYP3A4 expression. Several *PXR* polymorphisms have been described to date, but only a few have an effect on CYP3A4 function. Among them, we found rs2472677 and rs7643645. The rs7643645 SNP is located in the HNF4 binding site of the promoter of the *PXR* gene and has been associated with decreased PXR and CYP3A4 mRNA levels as well as CYP3A4 activity. The rs2472677 variant is located in the HNF3β binding site of the same promoter and results in increased PXR mRNA levels as well as basal CYP3A4 activity [21]. For practical purposes, in the present study the rs7643645 variant will be called *PXR-HNF4*/G or *PXR-HNF4/WT* and the rs2472677 variant will be called *PXR-HNF3β*/T or *PXR-HNF3β*/WT.

So far, there have been no reports in the literature exploring the possible association between *PXR* polymorphisms and PCa. Therefore, we performed a case-control study to investigate whether CYP3A4*1B, *PXR-HNF4/G*, and *PXR-HNF3β*/T allele variants are associated with a risk of developing PCa in Mexican men.

## Materials and Methods

### Study population

The present study used a hospital-based case-control design. The case group was recruited from the National Institute of Cancer and included 99 patients with a histologically confirmed diagnosis of PCa. Clinical characteristics, such as Gleason grade, prostate-specific antigen (PSA) levels at the time of diagnosis, digital rectal examination (DRE; according to the American Urological Association recommendations), tumor lymph node metastasis (TNM) [22], and age at the time of diagnosis were obtained from medical records. Categories of clinical characteristics were defined as follows: PSA two groups, (cut point, 10 ng/mL); Gleason grade two groups (cut point, 7); and TNM two groups (TNM≤2 and TNM≥3) [15]. The control group consisted of 144 patients with no history of any cancer, including prostate cancer, with a PSA<4.0 ng/mL, normal DRE, and was recruited from the Juárez Hospital. All subjects were unrelated men (self-reporting) between 60 and 76 years of age. The National Institute of Cancer and the Juárez Hospital Ethics Committees approved the present study and written informed consent was obtained from all subjects. The complete clinical and genetic per-patient data can be found in the supporting information (Table S1 and S2).

### DNA extraction

Genomic DNA was isolated from 5 ml of whole blood using a sodium perchlorate/chloroform extraction method. Briefly, DNA was prepared by combining each blood sample with 35 mL of lysis buffer [320 mM sucrose, 5 mM MgCl_2_, 1% (v/v) Triton X-100, 10 mM Tris-HCl, pH 8]. The nuclear pellet was collected by centrifugation at 2000×g for 10 min and then resuspended in 2 mL of solution B [150 mM NaCl, 60 mM EDTA, 1% (w/v) sodium dodecyl sulfate, 400 mM Tris-HCl, pH 8]. The suspension was mixed with 0.5 mL of 5 M sodium perchlorate and then incubated at 65 °C for 30 min. Following the incubation, 2 mL of chloroform was added, and the mixture was centrifuged at 1400×g for 10 min. The aqueous DNA-containing upper phase was precipitated by addition of 2 volumes of 100% ethanol and washed with 70% ethanol. The DNA was then resuspended in 200 µL of 10 mM Tris-HCl, 1 mM EDTA, pH 7.4, and quantified by measuring absorbance at a wavelength of 260 nm.

### Genotyping

Genotyping of CYP3A4*1B, *PXR-HNF3β*, and *PXR-HNF4* was conducted by real-time PCR using a StepOne Real-Time PCR System with TaqMan Universal PCR Master Mix (Applied Biosystems, USA). PCR was conducted using 5 µl of TaqMan Universal PCR Master Mix, 0.25 µl of primer-probe mix (containing 36 µM of each primer and 8 µM of dye-labeled probe), and 20 µg of DNA template. The final reaction volume was brought to 10 µl with H_2_O. Forty PCR cycles of the following parameters were used: initial denaturalization at 95°C for 10 min, 15 s at 92°C, and then 60°C for 1 min. After each amplification an allelic discrimination was made to determine the genotype of each subject. The primers and probes sequences used for *CYP3A4* were as follows: 5′-TGGAATGAGGACAGCCATAGAGA-3′ (forward), 5′-AGTGGAGCCATTGGCATAAAATCT-3′ (reverse), *1A probe (VIC): AAGGGCAAGAGAGAG, and *1B probe (FAM): AAGGGCAGGAGAGAG. For *PXR-HNF3β*: 5′- GCACAAACATTTTCAATTTCAATGAAGTTCA-3′ (forward), 5′- CATTCGGAAGACTTATTCTATTCCTGTCT-3′ (reverse), *PXR-HNF3β/WT* probe (VIC): CCATATTTTTTCTGATTAAA, and *PXR-HNF3β/T* probe (FAM): CCATATTTTTTTTGATTAAA. For *PXR-HNF4*: 5′-CACCATGCTTAGCTACAGCTCTATT-3′ (forward), 5′- GGCAAGATCACAACATGGGAAGA-3′ (reverse), *PXR-HNF4/WT* probe (VIC): AAAATGGCCTGTGGTCC, and *PXR-HNF4/G* probe (FAM): TGGCCCGTGGTCC.

### Statistical analysis

Statistical analysis was conducted using the STATA statistical package (version 10.1, STATA Corp., College Station, TX). T-test, *X^2^* test or Fisher's exact test were used to evaluate whether the distribution of genotype frequencies of CYP3A4*1B, *PXR-HNF4*, and *PXR-HNF3β* varied among cases and controls. For the comparison of clinical characteristics in the case group, ORs were calculated as an estimate of relative risk and 95% confidence intervals (CIs) were calculated using a bivariate logistic model. A value of p<0.03 was considered statistically significant after multiple testing adjustment by using the Bonferroni correction. The *X^2^* test was also used to assess deviations of allelic frequencies from Hardy-Weinberg equilibrium. The interaction between alleles was analyzed using the software Plink V 1.07. The number (n) of the case population for each association with clinical characteristics is indicated in the tables.

The sensitivity and specificity from significant models were estimated [23]. A cutoff of 50% for the classification of the event and the Receiver Operating Characteristic (ROC curve) were used. All calculations were performed using STATA as post logistic model estimation.

## Results

Clinical characteristics of the studied subjects were obtained from medical records and are presented in Table 1. No differences were observed in marital status or age between the two groups. As expected, prostate cancer patients presented significantly higher levels of PSA than in the control group (167 vs. 1.73 ng/mL, respectively) as well as a higher score for DRE grade III.

**Table 1:** Characteristics of the study subjects.

	No. of subjects (%)		
	Case	Control	p value^a^
Residence area			
South central	71 (76.34)	123 (88.49)	**0.01** *
North central	3 (3.23)	2 (1.44)	0.392&
Southeast	7 (7.52)	2 (1.44)	0.032&
Northeast	1 (1.08)	0 (0.0)	0.401&
East	9 (9.68)	10 (7.19)	0.49*
West	2 (2.15)	2 (1.44)	0.999&
Marital status			
Married	71 (71.71)	95 (68.84)	0.47*
Divorced	4 (4.04)	8 (5.80)	0.203&
Cohabitation	8 (8.09)	8 (5.80)	0.46*
Single	4 (4.04)	15 (10.87)	0.087&
Widow	12 (12.12)	12 (8.70)	0.35*
Age (Median ± S.D.)α	67.56±8.3	68.61±7.9	0.25*
Clinical characteristics			
PSA (Median ± S.D.)α	167.36±589.64	1.73±1.03	**0.001** *
DRE			
Grade I	8 (19.51)	38 (29.93)	0.17*
Grade II	21 (51.22)	79 (62.20)	0.23*
Grade IIIs	12 (29.27)	10 (7.87)	**0.0004** *

PSA, prostate specific antigen. DRE, digital rectal examination.

^*^x^2^ test.

& Fisher exact when expected value<5.

α p values were obtained using t^−^ test or one way ANOVA test as applied.

### Analysis between case and control groups

The genotype frequencies of *CYP3A4* and *PXR* between prostate cancer patients and controls are shown in Table 2. Alleles for *PXR* were in Hardy-Weinberg equilibrium (HWE), but CYP3A4*1B was only in HWE in the control group (data not shown). The CYP3A4*1B/*1B genotype was only present in prostate cancer patients. No differences were observed for CYP3A4*1A/*1B or CYP3A4*1A/*1A genotypes between both groups. Moreover, when *PXR* polymorphisms were compared between case and control groups, no differences in genotype frequencies for either *PXR-HNF3β* or *PXR-HNF4* alleles were observed.

**Table 2:** Distribution of *CYP3A4* and *PXR* genotypes.

Genotype	Case (n, %)	Control (n, %)	p value
* CYP3A4*			
* * * *1A/* * *1A*	83 (83.83)	116 (80.56)	0.51*
* * * *1A/* * *1B*	12 (12.12)	28 (19.44)	0.13*
* * * *1B/* * *1B*	4 (4.04)	0 (0)	**0.01** &
* PXR-HNF3β*			
* WT/WT*	40 (40.40)	50 (34.72)	0.36*
* WT/T*	43 (43.43)	72 (50.0)	0.31*
* T/T*	16 (16.16)	22 (15.28)	0.85*
* PXR-HNF4*			
* WT/WT*	21 (21.21)	26 (18.06)	0.54*
* WT/G*	45 (45.45)	75 (52.08)	0.30*
* G/G*	33 (33.33)	43 (29.86)	0.56*

^*^x^2^ test.

& Fisher's exact test.

n, No. of subjects.

We then determined the distribution of the combined *CYP3A4* and *PXR* allelic variants. Individuals with the combined genotypes CYP3A4*1B/*1B and *PXR-HNF4G/G*, or CYP3A4*1B/*1B and *PXR-HNF3β*WT/WT, were only present in the case group. For the other combinations, no differences were observed between control and case groups (Table 3).

**Table 3:** Distribution of combined *CYP3A4* and *PXR* allelic variants.

				*CYP3A4*					
	* *1A/* * *1A* (n, %)			* *1A/* * *1B* (n, %)			* *1B/* * *1B* (n, %)		
Genotype	Case	Control	p value	Case	Control	p value	Case	Control	p value
* PXR-HNF3β*									
* WT/WT*	34 (34.34)	40 (27.78)	0.27*	2 (2.02)	10 (6.94)	0.08&	3 (3.03)	0 (0.0)	**0.03** &
* WT/T*	35 (35.35)	59 (40.97)	0.37*	8 (8.08)	13 (9.02)	0.79*	1 (1.01)	0 (0.0)	0.22&
* T/T*	14 (14.14)	17 (11.80)	0.59*	2 (2.02)	5 (3.47)	0.50&	0 (0.0)	0 (0.0)	NA
* PXR-HNF4*									
* WT/WT*	16 (16.16)	21 (14.58)	0.73*	5 (5.05)	5 (3.47)	0.54*	0 (0.0)	0 (0.0)	NA
* WT/G*	40 (40.40)	59 (40.98)	0.92*	5 (5.05)	16 (11.11)	0.09*	0 (0.0)	0 (0.0)	NA
* G/G*	27 (27.27)	36 (25.00)	0.69*	2 (2.02)	7 (4.86)	0.24&	4 (4.04)	0 (0.0)	**0.01** &

^*^x^2^ test.

& Fisher's exact test. NA, not applicable.

n, No. of subjects.

### Analysis among cases

**Figure 1:**
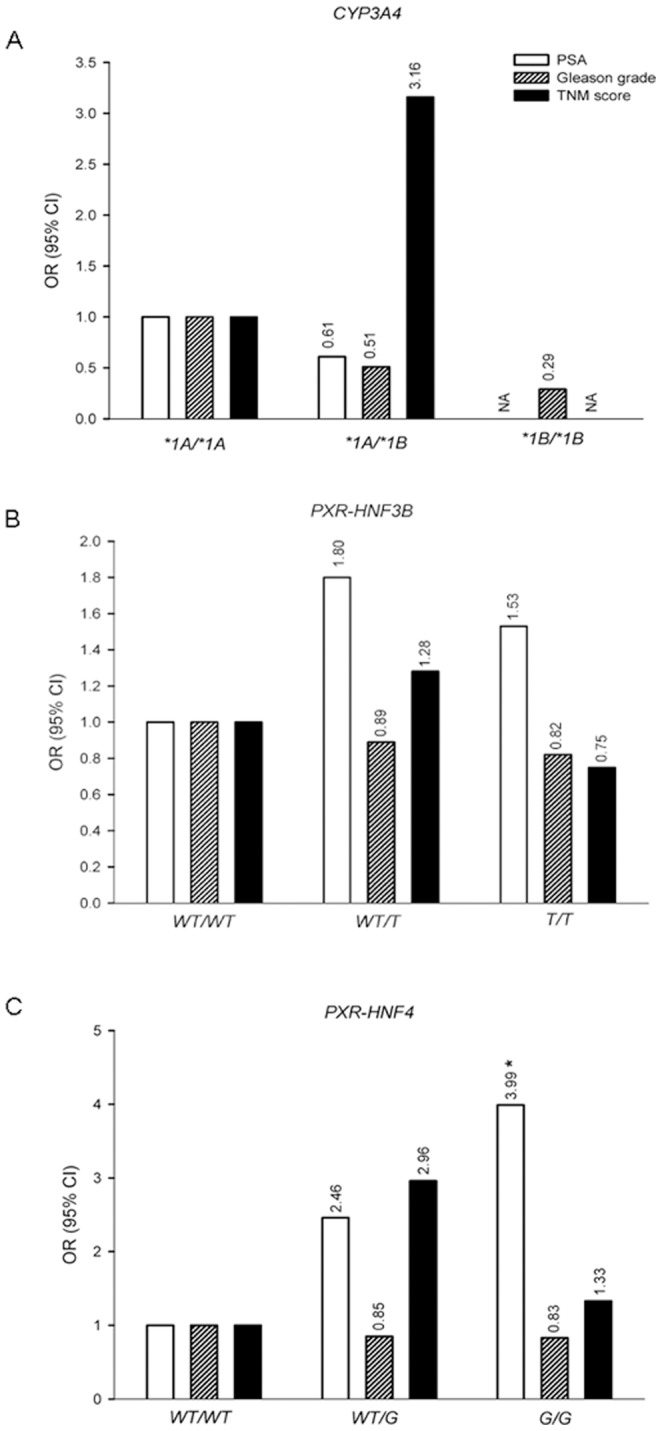
Association between *CYP3A4* (A), *PXR-HNF3β* (B), and *PXR-HNF4* (C) genotypes and clinical characteristics among cases. Case group was categorized as follows: PSA two groups, (cut point, 10 ng/mL); Gleason grade two groups (cut point, 7); and TNM two groups (TNM≤2 and TNM≥3). ORs were calculated as an estimate of relative risk and 95% confidence intervals (CIs) were calculated using a bivariate logistic model. PSA, prostate-specific antigen. TNM, tumor lymph nodes metastasis. *p = 0.03.

**Figure 2:**
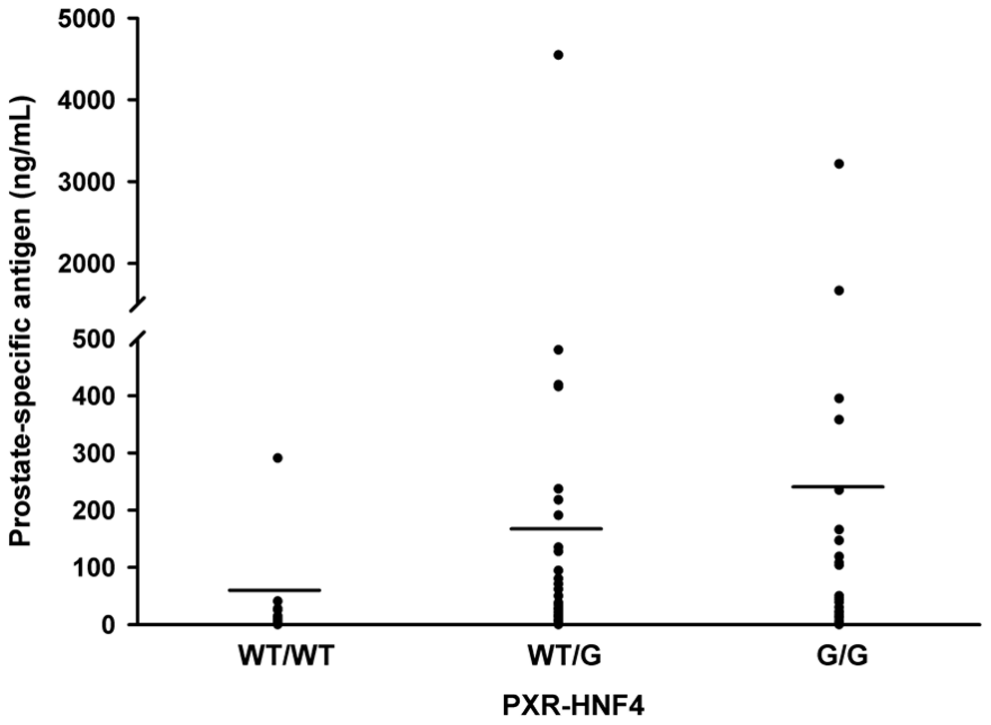
*PXR-HNF4*/G variant is associated with higher PSA levels. Individuals from the case group were genotyped for *PXR-HNF4*/G variant and divided in groups according to their genotype: 20 individuals corresponded to homozygous *PXR-HNF4-WT/WT*, 45 corresponded to heterozygous *PXR-HNF4-WT/G*, and 28 corresponded to *PXR-HNF4-G/G*. The data were analyzed by using the Mann Whitney U test. Horizontal line indicates the median. *WT/WT vs WT/G*, p = 0.05; *WT/WT vs G/G*, p = 0.02.

**Figure 3:**
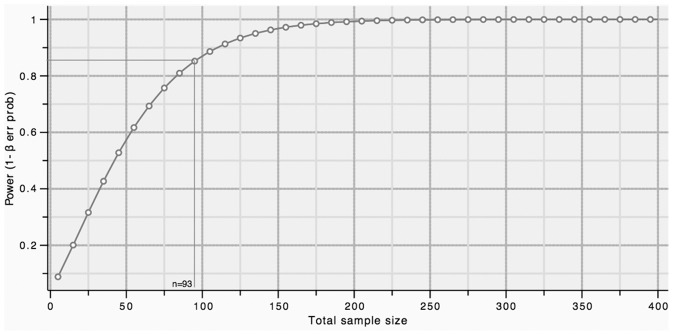
A power analysis for *PXR-HNF4* polymorphism was calculated considering an effect size (OR) equal to 3.9, a balanced design (p = 0.5) with equal sample frequencies for X = 0 and X = 1, in a two-sided test with α = 0.05. It was also considered the pseudo R2, logistic model (R2 = 0.0432). For a sample size of 93 individuals the power calculated was 0.84.

Stratification of the *CYP3A4* and *PXR* genotypes and PSA, Gleason grade, and TNM score among prostate cancer patients are shown in Figure 1 and Table S3. There were no differences in the *CYP3A4* genotype by comparison of PSA or Gleason grade. Twenty five percent of the prostate cancer patients with a TNM≥3 were heterozygous for the *CYP3A4* variant and had an OR of 3.16 compared to the *1A/*1A genotype, but this difference was not statistically significant (p = 0.10) (Table S3). For the *PXR-HNF4* genotype, an association of *PXR-HNF4*/G variant with higher PSA levels was observed in case group (Figures 1 and 2). Individuals with PSA>10 ng/ml were *WT/G* (49.2%) and *G/G* (34.92%) with ORs of 2.46 (p = 0.10) and 3.99 (p = 0.03), respectively. On the other hand, sixty two percent of the cases with a TNM≥3 were heterozygous for the *PXR-HNF4* allele with an OR of 2.96, but this result was not statistically significant. No associations were observed for *PXR-HNF4* genotypes with the Gleason grade. The PSA, Gleason grade, and TNM score values were not associated with the *PXR-HNF3β* genotype among PCa patients. In order to determine the power of the present study for *PXR-HNF4* polymorphism a power analysis was performed, and a 1-β value of 84 was obtained (Figure 3).

A stronger association between the *CYP3A4* genotype and Gleason and TNM grades in patients diagnosed at the age of 65 years or older has been reported previously [15]. Therefore, we investigated the association of *CYP3A4* and *PXR-HNF4* genotypes with clinical characteristics among individuals diagnosed at the age of 65 years or older. In order to compare the results with those previously reported, a dominant model was used (i.e. WT versus heterozygous and homozygous for the variant).

No association between *CYP3A4* genotypes and PSA or Gleason grade were observed. However, men with *1B allele showed an increased risk of having a higher TNM score (OR = 2.2), but the statistical analysis indicated that this association was not significant (Table 4). Table 5 shows ORs comparing *PXR-HNF4*-*WT* to *PXR-HNF4 WT/G* and *PXR-HNF4 -G/G* in combination (dominant model). The data indicate that men with the G variant showed an increased risk of having higher PSA levels (OR = 10.8; p = 0.006). Sensitivity and specificity analysis indicate that 84% of individuals over 65 years old with WT/G or G/G genotype will have a high risk to present higher levels of PSA.

**Table 4:** Association of *CYP3A4* genotype and clinical characteristics in patients diagnosed at the age of ≥65.

Clinic characteristics	*CYP3A4* genotype, n (%)			OR (95% CI) a	*p value
	* *1A/* * *1A*	* *1A/* * *1B*	* *1B/* * *1B*		
PSA at diagnosis (n = 41)					
≤10	7 (77.78)	1 (11.11)	1 (11.11)	1.0	
>10	25 (78.13)	3 (9.37)	4 (12.50)	1.0 (0.17-6.05)	0.98
Gleason grade (n = 36)					
≤7	11 (73.33)	1 (6.67)	3 (20.00)	1.0	
>7	17 (80.96)	2 (9.52)	2 (9.52)	1.5 (0.31-7.50)	0.58
TNM grade (n = 33)					
≤2	22 (81.48)	1 (3.70)	4 (14.82)	1.0	
≥3	4 (66.68)	1 (16.66)	1 (16.66)	2.2 (0.31-15.54)	0.42

PSA, prostatic-specific antigen. TNM, tumor lymph nodes metastasis.

a Comparing those with *1A /* *1A versus* those with

*1B /* *1B* and *1A /* *1B* genotypes (dominant model).^*^ Logistic model.

n, No. of subjects.

**Table 5:** Association of *PXR-HNF4* genotype and clinical characteristics in patients diagnosed at the age of ≥65.

Clinic characteristics	*PXR-HNF4* genotype, n (%)			OR (95% CI) ^a^	^*^p value
	*WT/WT*	*WT/G*	*G/G*		
PSA at diagnosis (*n* = 41)					
≤10	6 (66.66)	2 (22.22)	1 (11.11)	1.0	
>10	5 (15.63)	15 (46.87)	12 (37.50)	10.8 (2.00-58.10)	**0.006**
Gleason grade (*n* = 36)					
≤7	4 (26.67)	5 (33.33)	6 (40.00)	1.0	
>7	6 (28.57)	9 (42.86)	6 (28.57)	0.9 (0.20-4.01)	0.90
TNM score (*n* = 33)					
≤2	7 (25.92)	10 (37.04)	10 (37.04)	1.0	
≥3	2 (33.33)	3 (50.00)	1 (16.67)	0.7 (0.10-4.69)	0.71

PSA, prostatic-specific antigen. TNM, tumor lymph nodes metastasis.

^a^Comparing those with *WT versus* those with *G/G* and *WT* ***/*** *G* genotypes (dominant model).

^*^Logistic model.

n, No. of subjects.

No associations with Gleason grade and TNM score were identified. Taken together, these results clearly indicate that PXR-*HNF4/G* polymorphisms increase the risk of having higher PSA levels among cancer prostate patients.

## Discussion

The etiology of prostate cancer still remains unclear and involves several factors, including genetic determinants. Therefore, the identification of genetic risk factors for prostate cancer susceptibility is important. Many studies have focused on *CYP3A4* gene polymorphisms because this enzyme participates in testosterone metabolism. In the present case-control study, we investigated whether CYP3A4*1B variant was associated with several clinic characteristics of prostate cancer. The CYP3A4*1B variant was not in HWE in the case group most likely due to the excess of homozygous CYP3A4*1B genotype presented in this group. Deviation from HWE should not be unexpected, particularly when an allele is associated with a disease, which is the case in this study. In fact, similar studies show data that do not meet the Hardy-Weinberg population laws [14], [15], [24]. Although the CYP3A4*1B/*1B genotype is overrepresented in prostate cancer patients, no differences were observed in the frequency of this allele between controls and cases (Table S4). Moreover, no association of this polymorphism with PSA, Gleason grade, or TNM was observed. On the other hand, it has been reported that the incidence rate of PCa is higher in patients with benign prostatic hyperplasia having the CYP3A4*1B allele compared to those having the CYP3A4*1A allele [25]. Considering the above, it would be interesting to assess whether this association is also present in the Mexican population. Another important consideration is the potential clinical impact of the CYP3A4*1B variant on the individual response to hormonal therapy.

A previous study reported a stronger association between the CYP3A4*1B genotype and Gleason grade and TNM score in patients diagnosed at the age of 65 years or older [14], [15]. In the present patients population, the dominant model analysis showed that individuals diagnosed at the age of 65 years or older and with the CYP3A4*1B allele had a higher risk of a TNM≥3 (OR = 2.2). However, this increase was not statistically significant. Moreover, the present study did not find an association between CYP3A4*1B polymorphisms and clinic characteristics of prostate cancer. Other studies have also failed to show an association between prostate cancer and this allelic variant [17]. The functional significance of the CYP3A4*1B polymorphism has been studied using *in vitro* and *in vivo* approaches. Transactivation studies indicate that the -290A/G variant results in an increase in reporter gene activity, suggesting that the CYP3A4*1B polymorphism is unlikely to decrease the capacity to inactivate testosterone and therefore increase the risk for PCa development [26] [27]. More controversial results regarding *in vivo* studies have been reported. Wandel and collaborators [19] showed a modest difference in CYP3A4 activity between African Americans and European Americans associated with the CYP3A4*1B frequency. However, Lamba and collaborators [28] did not find any association between this polymorphism and the CYP3A4 phenotype. Ball [29] reported similar results. The lack of a correlation could be explained by other genetic polymorphisms involved in hormone metabolism, such as those presented at the *CYP3A5* gene. Moreover, sequence variation of transcription factors that modulate the expression of CYP3A4 and other hormone metabolizing enzymes should be considered as well. In particular, PXR may play an important role, since several transporters and metabolizing enzymes, including CYP3A4, are under its transcriptional regulation. In the present study, neither *PXR-HNF4/G* nor *PXR-HNF3β*/T alleles were over represented in case group. However, a clear association between the *PXR-HNF4/G* allele and PSA levels was observed. We found that heterozygous and homozygous prostate cancer patients had a higher risk of presenting higher levels of PSA (OR = 2.46 and OR = 3.99, respectively). When the analysis was applied to patients diagnosed at the age of 65 years or older, the dominant model analysis showed that individuals with this allele presented an even higher risk of having high PSA levels ≥10 (OR = 10.8).

The *PXR-HNF4* (69789A>G) polymorphism modifies an HNF4 binding site located in the *PXR* gene promoter, which results in lower PXR and CYP3A4 mRNA levels together with a decrease in CYP3A4 basal activity [30]. On the other hand, it has been shown that prostate tissue from prostate cancer patients express lower levels of PXR and CYP3A4 proteins compared to benign prostate tissue from control group [31] [10]. This most likely favors the conversion of testosterone to dihydrotestosterone (DHT) increasing the risk of developing PCa as well as prostate cancer markers such as PSA. PSA is a member of the kallikrein gene family. Several data indicate that PSA is under androgenic regulation. Induction of PSA mRNA is driven by mibolerone and DHT and inhibited by antiandrogens, suggesting that this effect is androgen receptor-dependent [32] [33] [34]. Once PSA is secreted it degrades extracellular proteins facilitating the invasion of prostate cancer cells [35]. PSA has been widely used to screen men for PCa leading to a dramatic reduction in PCa death rates. However, its use is controversial due to its limit to distinguish between cancer and benign prostatic hyperplasia, or between indolent and aggressive cancer resulting in overdetection and overtreatment. The present results should be understood by considering the above.

In addition to CYP3A4, PXR regulates the expression of other genes involved in steroid hormone metabolism, such as CYP17 and CYP24A1, which might account for alterations in testosterone bioavailability [36] [37]. The *PXR-HNF4/G* allele frequency in the control group was 0.37, which is similar to that observed for Caucasians (0.36) [30]. In contrast, this allele frequency in the prostate cancer group was 0.56. However, this difference was not statistically significant. Nevertheless, the presence of the *PXR-HNF4/G* allele is high in both populations, Caucasian and Mexicans, and therefore it would be interesting to explore possible associations of this polymorphism and drug metabolism as well as other pathologies.

Interestingly, the patients homozygous for the CYP3A4*1B allele were also homozygous for the *PXR-HNF4/G* allele. Therefore, we investigated whether an epistasis phenomenon was involved and found that the two alleles did not interact.

In summary, the present study is the first to show that the presence of the *PXR-HNF4/G* allele increases the risk of having higher levels of PSA in patients with prostate cancer. It is also the first study that evaluates, in a Mexican population, the association of *CYP3A4* and *PXR* gene polymorphisms with PCa. In agreement with previous studies, the present data suggest that the CYP3A4*1B polymorphism appears to be a gene with low penetrance and therefore has moderate effects on the risk of developing prostate cancer. Finally, future studies should be performed to assess the PXR variants role in PCa development.

## Supporting Information

Table S1 **Clinical and genetic per–patient results (Cases).**
(DOC)

Table S2 **Clinical and genetic per–patient results (Controls).**
(DOCX)

Table S3 **Relation between** ***CYP3A4*** **and** ***PXR*** **genotypes and clinical characteristics among cases.**
(DOC)

Table S4 ***CYP3A4*** **and** ***PXR*** **allelic frequencies.**
(DOC)
