# Supplementary material for: The PXR rs7643645 Polymorphism Is Associated with the Risk of Higher Prostate-Specific Antigen Levels in Prostate Cancer Patients
Source: PLoS One. 2014 Jun 12;9(6):e99974. doi: 10.1371/journal.pone.0099974 (PMC4055777; doi:10.1371/journal.pone.0099974)
Supplement: Table S1 — Clinical and genetic per–patient results (Cases). (DOC) [file pone.0099974.s001.doc]

**Table S1. Clinical and genetic per–patient results (Cases)**

| ID | *CYP3A4*1B* Genotype | | *PXR-HNF3beta* Genotype | | *PXR-HNF4* Genotype | | Age | PSA | | Marital status | | | Residence area | DRE | Gleason score | | TNM | |  | |
| --- | --- | --- | --- | --- | --- | --- | --- | --- | --- | --- | --- | --- | --- | --- | --- | --- | --- | --- | --- | --- |
| P1 | (+/+) | | (+/+) | | (-/-) | | 65 | 1.14 | | Widow | | | South central | NA | 4 | | 1 | |  | |
| P2 | (+/+) | | (+/+) | | (-/-) | | 64 | 104 | | Cohabitation | | | NA | NA | 10 | | 1 | |  | |
| P3 | (+/+) | | (+/-) | | (+/-) | | 59 | 8.8 | | Married | | | South central | II | 2 | | 1 | |  | |
| P4 | (+/+) | | (-/-) | | (+/+) | | 62 | 9.6 | | Married | | | South central | III | 9 | | 1 | |  | |
| P5 | (+/-) | | (+/-) | | (+/-) | | 79 | 12.7 | | Married | | | South central | NA | NA | | NA | |  | |
| P6 | (+/+) | | (-/-) | | (+/-) | | 69 | 27 | | Married | | | South central | NA | 7 | | 2 | |  | |
| P7 | (+/+) | | (+/+) | | (-/-) | | 62 | 108 | | Married | | | South central | NA | 8 | | 1 | |  | |
| P8 | (+/+) | | (+/-) | | (+/+) | | 73 | 8.3 | | Widow | | | Southeast | II | 8 | | 2 | |  | |
| P9 | (+/+) | | (+/-) | | (+/-) | | 66 | 13.9 | | Married | | | NA | NA | 7 | | 3 | |  | |
| P10 | (+/+) | | (+/+) | | (+/+) | | 62 | 40.9 | | Married | | | Southeast | NA | 7 | | 1 | |  | |
| P11 | (+/+) | | (+/-) | | (+/-) | | 70 | 419 | | Widow | | | South central | II | 9 | | 2 | |  | |
| P12 | (+/+) | | (+/-) | | (+/-) | | 74 | 38.2 | | Married | | | South central | II | 8 | | 2 | |  | |
| P13 | (+/+) | | (+/-) | | (+/-) | | 71 | 14 | | Married | | | South central | II | 7 | | 2 | |  | |
| P14 | (+/+) | | (+/+) | | (+/-) | | 67 | 4.9 | | Married | | | NA | III | 8 | | 3 | |  | |
| P15 | (+/+) | | (+/+) | | (-/-) | | 72 | 14 | | Married | | | North central | NA | 6 | | NA | |  | |
| P16 | (+/+) | | (+/-) | | (+/-) | | 73 | 5.9 | | Married | | | South central | NA | 6 | | 1 | |  | |
| P17 | (+/-) | | (+/-) | | (+/-) | | 79 | 13 | | Cohabitation | | | South central | I | 9 | | 3 | |  | |
| P18 | (+/+) | | (+/+) | | (-/-) | | 62 | 15.1 | | Single | | | South central | NA | 6 | | 1 | |  | |
| P19 | (+/+) | | (+/+) | | (-/-) | | 62 | NA | | Married | | | South central | NA | 7 | | 1 | |  | |
| P20 | (+/+) | | (+/+) | | (+/-) | | 63 | 28.8 | | Single | | | South central | II | 8 | | 2 | |  | |
| P21 | (+/-) | | (+/-) | | (+/+) | | 49 | 0.24 | | Cohabitation | | | South central | III | 4 | | 2 | |  | |
| P22 | (+/+) | | (+/-) | | (+/-) | | 73 | 8.4 | | Married | | | East | II | 6 | | 2 | |  | |
| P23 | (-/-) | | (+/+) | | (-/-) | | 79 | 43.7 | | Married | | | South central | III | 7 | | 2 | |  | |
| P24 | (+/+) | | (-/-) | | (+/+) | | 73 | 5.6 | | Cohabitation | | | NA | NA | 7 | | 2 | |  | |
| P25 | (+/+) | | (+/-) | | (+/+) | | 64 | 5.4 | | Married | | | South central | I | 8 | | 1 | |  | |
| P26 | (+/+) | | (+/+) | | (-/-) | | 72 | 0.2 | | Married | | | South central | III | NA | | 1 | |  | |
| P27 | (+/+) | | (+/+) | | (-/-) | | 56 | 119 | | Married | | | NA | I | 10 | | 1 | |  | |
| P28 | (+/+) | | (+/-) | | (+/-) | | 62 | 17.4 | | Married | | | East | NA | 8 | | 1 | |  | |
| P29 | (+/+) | | (+/-) | | (+/-) | | 63 | 94.5 | | Cohabitation | | | South central | II | 9 | | 2 | |  | |
| P30 | (+/+) | | (+/-) | | (+/-) | | 65 | 22.4 | | Married | | | Southeast | NA | 6 | | 1 | |  | |
| P31 | (+/+) | | (+/-) | | (+/-) | | 66 | 8.9 | | Married | | | East | NA | 7 | | 3 | |  | |
| P32 | (+/+) | | (+/-) | | (+/-) | | 69 | 4546 | | Married | | | Southeast | NA | 9 | | 1 | |  | |
| P33 | (+/+) | | (+/+) | | (+/-) | | 66 | 2.6 | | Married | | | South central | NA | 6 | | 3 | |  | |
| P34 | (+/+) | | (-/-) | | (+/+) | | 74 | 2.5 | | Married | | | South central | NA | 8 | | 2 | |  | |
| P35 | (+/+) | | (+/-) | | (+/-) | | 60 | 5.7 | | Single | | | South central | II | 7 | | 1 | |  | |
| P36 | (+/-) | | (+/-) | | (+/-) | | 80 | 237 | | Married | | | South central | II | 9 | | 2 | |  | |
| P37 | (+/+) | | (+/+) | | (+/+) | | 72 | 696 | | Married | | | South central | NA | 8 | | 2 | |  | |
| P38 | (+/+) | | (+/-) | | (+/-) | | 78 | 480 | | Married | | | Southeast | NA | 7 | | NA | |  | |
| P39 | (+/-) | | (+/+) | | (-/-) | | 55 | 1.58 | | Married | | | South central | NA | 9 | | 3 | |  | |
| P40 | (+/-) | | (+/-) | | (+/+) | | 46 | 13.4 | | Married | | | South central | NA | 6 | | 1 | |  | |
| P41 | (+/-) | | (+/+) | | (-/-) | | 65 | 4 | | Cohabitation | | | South central | II | 5 | | 1 | |  | |
| P42 | (-/-) | | (+/+) | | (-/-) | | 55 | 15.2 | | Single | | | South central | III | 6 | | 1 | |  | |
| P43 | (+/+) | | (+/+) | | (+/-) | | 79 | 128 | | Widow | | | South central | NA | 6 | | 2 | |  | |
| P44 | (+/+) | | (+/+) | | (-/-) | | 73 | 46.8 | | Married | | | South central | NA | 9 | | 1 | |  | |
| P45 | (+/-) | | (-/-) | | (+/+) | | 50 | 25.7 | | NA | | | East | NA | 7 | | 1 | |  | |
| P46 | NA | | NA | | NA | | 74 | 0.56 | | Cohabitation | | | South central | NA | 3 | | 1 | |  | |
| P47 | (+/+) | | (+/-) | | (+/-) | | 63 | 38.23 | | Married | | | South central | NA | 6 | | 2 | |  | |
| P48 | (-/-) | | (+/+) | | (-/-) | | 79 | 395 | | Married | | | South central | II | 7 | | 2 | |  | |
| P49 | (+/+) | | (+/+) | | (-/-) | | 69 | 23 | | Married | | | South central | NA | 7 | | 3 | |  | |
| P50 | (+/-) | | (-/-) | | (+/+) | | 76 | 3.6 | | Married | | | South central | NA | 6 | | NA | |  | |
| P51 | (+/+) | | (+/-) | | (+/-) | | 52 | 191 | | Married | | | South central | NA | 9 | | NA | |  | |
| P52 | (+/-) | | (+/-) | | (+/-) | | 60 | 5.55 | | Married | | | Southeast | NA | 7 | | 3 | |  | |
| P53 | (+/+) | | (-/-) | | (+/+) | | 58 | 5.44 | | Married | | | South central | NA | 6 | | 1 | |  | |
| P54 | (+/+) | | (-/-) | | (+/+) | | 76 | 12.3 | | Married | | | South central | I | 8 | | 1 | |  | |
| P55 | (+/+) | | (+/+) | | (-/-) | | 67 | 18.1 | | Married | | | South central | NA | NA | | 1 | |  | |
| P56 | (+/+) | | (+/-) | | (+/-) | | 65 | 0.8 | | Cohabitation | | | South central | NA | 6 | | 1 | |  | |
| P57 | (+/+) | | (+/+) | | (-/-) | | 56 | 147 | | Married | | | South central | III | NA | | NA | |  | |
| P58 | (+/+) | | (+/-) | | (+/-) | | 64 | 7.4 | | Married | | | South central | NA | 8 | | 2 | |  | |
| **Table S1. (Cont.)** | | | | | | |  |  | |  | | |  |  |  | |  | |  | |
|  |  | |  | |  | |  |  | |  | | |  |  |  | |  | |  | |
| ID | *CYP3A4*1B* Genotype | | *PXR-HNF3beta* Genotype | | *PXR-HNF4* Genotype | | Age | PSA | | Marital status | | | Residence area | DRE | Gleason score | | TNM | |  | |
| P59 | (+/+) | | (+/+) | | (+/-) | | 69 | 6.56 | | Married | | | South central | NA | 10 | | 3 | |  | |
| P60 | (+/+) | | (+/-) | | (+/-) | | 76 | 50.1 | | Married | | | South central | II | 8 | | 2 | |  | |
| P61 | (+/+) | | (+/+) | | (-/-) | | 65 | 18 | | Divorced | | | South central | NA | 7 | | 2 | |  | |
| P62 | (+/+) | | (+/-) | | (+/-) | | 66 | 135 | | Married | | | South central | NA | 8 | | 3 | |  | |
| P63 | (+/+) | | (+/-) | | (+/+) | | 63 | 12.9 | | Married | | | South central | NA | 9 | | 2 | |  | |
| P64 | (+/+) | | (+/-) | | (+/-) | | 75 | 71 | | Widow | | | Southeast | III | 9 | | 2 | |  | |
| P65 | (+/+) | | (+/+) | | (+/-) | | 76 | 37.7 | | Married | | | South central | III | 8 | | 3 | |  | |
| P66 | (+/+) | | (+/-) | | (+/-) | | 63 | 416 | | Married | | | South central | III | 10 | | 3 | |  | |
| P67 | (+/+) | | (-/-) | | (-/-) | | 84 | 3216 | | Married | | | East | II | 9 | | 2 | |  | |
| P68 | (+/+) | | (+/-) | | (+/-) | | 68 | 28 | | Married | | | Northeast | NA | 6 | | NA | |  | |
| P69 | (+/+) | | (+/-) | | (+/-) | | 64 | 20.4 | | Married | | | South central | I | 6 | | 1 | |  | |
| P70 | (+/+) | | (+/+) | | (-/-) | | 75 | 12.1 | | Married | | | South central | I | 7 | | 2 | |  | |
| P71 | (+/+) | | (+/-) | | (+/+) | | 68 | 27.7 | | Married | | | North central | NA | 7 | | 2 | |  | |
| P72 | (+/+) | | (+/-) | | (+/-) | | 73 | 61.8 | | Married | | | South central | NA | 9 | | 1 | |  | |
| P73 | (-/-) | | (+/-) | | (-/-) | | 73 | 166 | | Widow | | | NA | II | 8 | | 2 | |  | |
| P74 | (+/+) | | (+/+) | | (-/-) | | 67 | 235 | | Married | | | East | NA | 9 | | NA | |  | |
| P75 | (+/+) | | (+/+) | | (-/-) | | 73 | NA | | Married | | | South central | II | 9 | | 2 | |  | |
| P76 | (+/+) | | (-/-) | | (+/+) | | 72 | 291 | | Married | | | South central | III | 8 | | 3 | |  | |
| P77 | (+/-) | | (+/-) | | (+/-) | | 63 | 34.5 | | Married | | | South central | NA | 4 | | 2 | |  | |
| P78 | (+/+) | | (-/-) | | (+/+) | | 62 | 8.69 | | Married | | | South central | I | 9 | | 2 | |  | |
| P79 | (+/+) | | (+/+) | | (+/-) | | 71 | 25.9 | | Married | | | South central | NA | 6 | | 1 | |  | |
| P80 | (+/+) | | (-/-) | | (-/-) | | 74 | 50 | | Married | | | South central | II | 10 | | 3 | |  | |
| P81 | (+/+) | | (+/-) | | (-/-) | | 67 | 1.6 | | NA | | | East | NA | 4 | | 2 | |  | |
| P82 | (+/+) | | (-/-) | | (+/-) | | 75 | 16.4 | | Married | | | South central | NA | 7 | | 1 | |  | |
| P83 | (+/-) | | (+/-) | | (+/+) | | 59 | 26.1 | | Married | | | West | III | 9 | | 3 | |  | |
| P84 | (+/+) | | (+/+) | | (-/-) | | 69 | 30 | | Divorced | | | South central | NA | 9 | | 2 | |  | |
| P85 | (+/+) | | (+/+) | | (+/-) | | 63 | 13.8 | | Married | | | South central | II | 6 | | 2 | |  | |
| P86 | (+/+) | | (+/+) | | (+/-) | | 62 | 14.9 | | Married | | | North central | NA | NA | | NA | |  | |
| P87 | (+/+) | | (-/-) | | (+/+) | | 55 | 14.9 | | Married | | | South central | NA | 7 | | 1 | |  | |
| P88 | (+/+) | | (+/+) | | (+/+) | | 58 | 1.13 | | Married | | | South central | NA | NA | | NA | |  | |
| P89 | (+/+) | | (+/-) | | (-/-) | | 57 | 38.9 | | Married | | | South central | NA | NA | | 3 | |  | |
| P90 | (+/+) | | (-/-) | | (-/-) | | 70 | 358 | | Widow | | | South central | NA | 7 | | 1 | |  | |
| P91 | (+/+) | | (+/-) | | (-/-) | | 79 | 358 | | Widow | | | South central | II | 7 | | 2 | |  | |
| P92 | (+/+) | | (-/-) | | (+/+) | | 91 | NA | | Widow | | | East | NA | NA | | NA | |  | |
| P93 | (+/+) | | (+/+) | | (-/-) | | 68 | 6.05 | | Widow | | | South central | NA | NA | | NA | |  | |
| P94 | (+/+) | | (+/+) | | (-/-) | | 89 | 21.6 | | Widow | | | South central | II | 9 | | 2 | |  | |
| P95 | (+/+) | | (+/+) | | (-/-) | | 64 | 1667 | | Widow | | | East | II | NA | | 1 | |  | |
| P96 | (+/+) | | (+/+) | | (-/-) | | 49 | 0.56 | | Married | | | South central | NA | 8 | | NA | |  | |
| P97 | (+/+) | | (+/-) | | (+/-) | | 69 | 80.3 | | Married | | | South central | NA | 10 | | NA | |  | |
| P98 | (+/+) | | (+/+) | | (+/-) | | 65 | 2.4 | | Married | | | South central | I | 10 | | 1 | |  | |
| P99 | (+/+) | | (+/-) | | (+/-) | | 73 | 0.06 | | Married | | | West | NA | NA | | NA | |  | |
| P100 | (+/+) | | (+/-) | | (+/-) | | 77 | 218 | | Cohabitation | | | South central | NA | NA | | NA | |  | |
|  | |  | |  | |  | | |  | |  |  | |  | |  | |  | |  |

(+/+) = Wild-type variant, homozygous.

(+/-) = Heterozygous.

(-/-) = Polymorphic variant, homozygous.

NA, low DNA quality to perform analysis and/or missing data from medical records.
