# Supplementary material for: The PXR rs7643645 Polymorphism Is Associated with the Risk of Higher Prostate-Specific Antigen Levels in Prostate Cancer Patients
Source: PLoS One. 2014 Jun 12;9(6):e99974. doi: 10.1371/journal.pone.0099974 (PMC4055777; doi:10.1371/journal.pone.0099974)
Supplement: Table S2 — Clinical and genetic per–patient results (Controls). (DOCX) [file pone.0099974.s002.docx]

**Table S2. Clinical and genetic per–patient results (Controls)**

| ID | *CYP3A4*1B* Genotype | *PXR-HNF3beta* Genotype | *PXR-HNF4* Genotype | Age | PSA | Marital status | Residence area | DRE |
| --- | --- | --- | --- | --- | --- | --- | --- | --- |
| P201 | (+/+) | (+/+) | (-/-) | 72 | 2.62 | Married | South central | II |
| P202 | (+/+) | (+/-) | (+/-) | NA | NA | NA | NA | NA |
| P203 | (+/+) | (+/-) | (+/+) | 68 | 2.6 | Widow | South central | II |
| P204 | (+/+) | (+/+) | (+/-) | 67 | 1.8 | Married | South central | NA |
| P205 | (+/+) | (+/-) | (+/-) | 80 | 0.02 | Married | South central | I |
| P206 | (+/+) | (+/-) | (+/+) | 60 | 1.84 | Cohabitation | South central | II |
| P207 | (+/+) | (+/-) | (-/-) | 66 | 1.3 | Married | South central | NA |
| P208 | (+/+) | (+/-) | (+/-) | 68 | 2.09 | Married | South central | II |
| P209 | (+/-) | (+/+) | (-/-) | 69 | 0.09 | Cohabitation | South central | I |
| P210 | (+/+) | (+/+) | (-/-) | 74 | 1.02 | Married | South central | II |
| P211 | (+/+) | (+/+) | (-/-) | 75 | 1.62 | Single | South central | II |
| P212 | (+/+) | (+/-) | (+/-) | 60 | 0.25 | Divorced | South central | II |
| P213 | (+/-) | (+/-) | (+/-) | 67 | 1 | Married | South central | I |
| P214 | (+/-) | (-/-) | (+/+) | 74 | 1.01 | Divorced | South central | II |
| P215 | (+/+) | (+/-) | (+/-) | 74 | 0.93 | Married | South central | II |
| P216 | (+/+) | (+/-) | (+/-) | 71 | 1.72 | Single | South central | II |
| P217 | (+/+) | (+/-) | (+/-) | 68 | 1.48 | Married | South central | II |
| P218 | (+/+) | (-/-) | (-/-) | 70 | 0.58 | Widow | South central | II |
| P219 | (+/+) | (+/-) | (+/-) | 68 | 3.45. | Married | South central | II |
| P220 | (+/+) | (-/-) | (+/-) | 63 | 3.6 | Married | South central | III |
| P221 | (+/+) | (+/+) | (-/-) | 76 | 3.44 | Married | South central | II |
| P222 | (+/+) | (+/+) | (+/-) | 67 | 4.08 | Single | South central | II |
| P223 | (+/+) | (+/-) | (+/-) | 70 | 0.58 | Married | South central | II |
| P224 | (+/+) | (-/-) | (+/+) | 83 | 0.51 | Widow | South central | I |
| P225 | (+/+) | (-/-) | (+/+) | 76 | 4.02 | Single | South central | II |
| P226 | (+/-) | (+/+) | (-/-) | 74 | 2.62 | Married | Southeast | II |
| P227 | (+/+) | (+/+) | (+/-) | 61 | 1.21 | Married | South central | I |
| P228 | (+/+) | (+/-) | (+/-) | 67 | 1.24 | Married | South central | I |
| P229 | (+/-) | (+/+) | (+/-) | 62 | 1.5 | Married | South central | I |
| P230 | (+/-) | (-/-) | (+/-) | 65 | 2.38 | Married | South central | II |
| P231 | (+/+) | (+/+) | (+/-) | 73 | 1.88 | Married | South central | I |
| P232 | (+/+) | (+/+) | (-/-) | 74 | 2.22 | Married | East | I |
| P233 | (+/-) | (+/+) | (-/-) | 74 | 1.98 | Married | South central | II |
| P234 | (+/-) | (+/-) | (+/-) | 65 | 2.07 | Married | South central | II |
| P235 | (+/-) | (+/-) | (+/-) | 62 | 0.79 | Married | South central | II |
| P236 | (+/+) | (+/+) | (-/-) | 112 | 3.26 | Widow | South central | II |
| P237 | (+/+) | (+/-) | (+/-) | 61 | 1.28 | Married | South central | II |
| P238 | (+/+) | (+/-) | (+/-) | 64 | 0.31 | Married | South central | II |
| P239 | (+/-) | (-/-) | (+/+) | 64 | 0.31 | Married | South central | II |
| P240 | (+/+) | (-/-) | (+/+) | 61 | 3.17 | Married | South central | II |
| P241 | (+/+) | (+/+) | (-/-) | 60 | 1.02 | Married | South central | I |
| P242 | (+/+) | (+/+) | (-/-) | 84 | 2.01 | Widow | South central | II |
| P243 | (+/+) | (+/-) | (-/-) | 61 | 0.45 | Married | South central | I |
| P244 | (+/+) | (+/-) | (+/-) | 60 | 0.303 | Married | South central | II |
| P245 | (+/+) | (+/-) | (+/-) | 64 | 0.8 | Cohabitation | East | I |
| P246 | (+/+) | (+/-) | (+/-) | 69 | 0.54 | Married | East | III |
| P247 | (+/+) | (+/-) | (+/-) | 67 | 0.32 | Married | South central | II |
| P248 | (+/+) | (+/-) | (+/-) | 77 | 1.5 | Married | South central | II |
| P249 | (+/+) | (+/-) | (-/-) | 75 | 1.48 | Married | South central | II |
| P250 | (+/+) | (+/+) | (-/-) | NA | NA | NA | NA | NA |
| P251 | (+/-) | (-/-) | (+/-) | 66 | 2.5 | Married | East | III |
| P252 | (+/-) | (+/-) | (+/+) | 73 | 3 | Widow | South central | II |
| P253 | (+/-) | (-/-) | (+/+) | 72 | 3.08 | Married | North central | I |
| P254 | (+/+) | (+/-) | (+/-) | 63 | 1.14 | Single | South central | I |
| P255 | (+/+) | (+/+) | (-/-) | 69 | 3.42 | Single | South central | III |
| P256 | (+/+) | (+/-) | (+/+) | 80 | 3 | Married | South central | II |
| P257 | (+/+) | (+/-) | (+/-) | 74 | 0.4 | Widow | South central | I |
| P258 | (+/+) | (+/-) | (+/-) | 62 | 2.68 | Cohabitation | South central | II |
| P259 | (+/+) | (+/-) | (+/-) | 62 | 1.38 | Married | South central | II |
| **Table S2. (Cont.)** | | | |  |  |  |  |  |
|  | | | |  |  |  |  |  |
| ID | *CYP3A4*1B* Genotype | *PXR-HNF3beta* Genotype | *PXR-HNF4* Genotype | Age | PSA | Marital status | Residence area | DRE |
| P260 | (+/+) | (-/-) | (+/+) | 73 | 0.87 | Married | South central | III |
| P261 | (+/+) | (+/+) | (-/-) | 61 | 2.06 | Married | South central | II |
| P262 | (+/+) | (+/+) | (-/-) | 69 | 1.36 | Married | South central | II |
| P263 | (+/+) | (+/+) | (-/-) | 64 | 1.79 | Married | West | I |
| P264 | (+/+) | (-/-) | (+/+) | 88 | 0.6 | Married | South central | II |
| P265 | (+/+) | (+/+) | (+/-) | 62 | 2.36 | Married | South central | NA |
| P266 | (+/+) | (-/-) | (+/-) | 70 | 2.03 | Single | South central | II |
| P267 | (+/+) | (+/-) | (+/-) | 64 | 1.6 | Married | South central | II |
| P268 | (+/+) | (+/+) | (+/-) | 75 | 2.39 | Divorced | South central | II |
| P269 | (+/+) | (+/-) | (+/+) | 70 | 2.8 | Married | South central | II |
| P270 | (+/+) | (+/+) | (-/-) | 67 | 0.62 | Married | South central | NA |
| P271 | (+/-) | (+/+) | (-/-) | 61 | 1.23 | Widow | South central | II |
| P272 | (+/-) | (+/+) | (+/-) | 70 | 1.12 | Single | South central | II |
| P273 | (+/+) | (+/-) | (+/-) | 60 | 1.12 | Married | South central | II |
| P274 | (+/+) | (+/-) | (+/-) | 62 | 0.72 | Married | South central | II |
| P275 | (+/+) | (+/+) | (+/-) | 83 | 0.013 | Divorced | South central | II |
| P276 | (+/+) | (+/-) | (+/+) | 86 | 3 | Single | South central | NA |
| P277 | (+/+) | (+/-) | (-/-) | 61 | 0.53 | Married | South central | I |
| P278 | (+/+) | (+/-) | (+/-) | 73 | 0.53 | Married | North central | I |
| P279 | (+/+) | (+/-) | (+/-) | 74 | 1.22 | Married | South central | I |
| P280 | (+/-) | (+/-) | (+/-) | 60 | 2.66 | Cohabitation | South central | III |
| P281 | (+/+) | (+/-) | (+/-) | 62 | 4.06 | Married | South central | II |
| P282 | (+/+) | (+/+) | (-/-) | 63 | 2.67 | Cohabitation | South central | II |
| P283 | (+/+) | (-/-) | (+/+) | 64 | 0.68 | Married | South central | II |
| P284 | (+/+) | (+/-) | (+/-) | 64 | 2.13 | Married | East | I |
| P285 | (+/-) | (+/-) | (+/-) | 64 | NA | Married | South central | NA |
| P286 | (+/-) | (+/-) | (+/-) | 73 | 2.75 | Widow | South central | II |
| P287 | (+/+) | (+/+) | (-/-) | 63 | 2.29 | Single | South central | NA |
| P288 | (+/+) | (+/+) | (-/-) | 78 | 0.209 | Widow | East | II |
| P289 | (+/+) | (+/+) | (-/-) | 64 | 1.66 | Married | South central | I |
| P290 | (+/+) | (+/-) | (+/-) | 84 | 2.36 | Divorced | South central | NA |
| P291 | (+/+) | (+/-) | (+/-) | 61 | 0.77 | Married | South central | II |
| P292 | (+/+) | (+/-) | (+/-) | 66 | 1.72 | Married | South central | I |
| P293 | (+/+) | (+/-) | (+/-) | 68 | 2.9 | Married | South central | I |
| P294 | (+/+) | (+/-) | (+/-) | 63 | 1.05 | Married | South central | II |
| P295 | (+/+) | (+/-) | (+/-) | 72 | 0.84 | Married | South central | NA |
| P296 | (+/+) | (+/+) | (+/-) | 69 | 2.8 | Married | South central | II |
| P297 | (+/+) | (-/-) | (+/+) | 61 | 0.4 | Divorced | South central | II |
| P298 | (+/+) | (+/-) | (+/-) | 66 | 1.69 | Married | South central | I |
| P299 | (+/+) | (-/-) | (+/+) | 64 | 0.78 | Married | East | NA |
| P300 | (+/+) | (+/-) | (+/-) | 66 | 0.48 | Married | South central | II |
| P301 | (+/-) | (+/-) | (+/-) | 63 | 2.54 | Widow | South central | II |
| P302 | (+/+) | (+/-) | (+/-) | 60 | 0.41 | Divorced | East | I |
| P303 | (+/+) | (+/-) | (+/+) | 62 | 2.86 | Married | South central | II |
| P304 | (+/+) | (+/-) | (+/-) | 63 | NA | Cohabitation | Southeast | III |
| P305 | (+/+) | (+/-) | (+/-) | 71 | 0.7 | Married | South central | NA |
| P306 | (+/-) | (+/+) | (-/-) | 65 | 1.91 | Married | South central | III |
| P307 | (+/+) | (+/-) | (+/-) | 83 | 2.3. | Married | South central | II |
| P308 | (+/+) | (-/-) | (+/+) | NA | NA | NA | NA | NA |
| P309 | (+/+) | (+/+) | (-/-) | 76 | 3.49 | Married | South central | II |
| P310 | (+/+) | (+/-) | (-/-) | 80 | 3.58 | Married | South central | II |
| P311 | (+/+) | (+/+) | (-/-) | 60 | 1.4. | Married | South central | III |
| P312 | (+/-) | (+/-) | (+/-) | 90 | 2.03 | Married | South central | I |
| P313 | (+/+) | (-/-) | (+/+) | 80 | 2.6 | Married | East | II |
| P314 | (+/+) | (+/+) | (+/-) | 66 | 0.86 | Married | South central | I |
| P315 | (+/-) | (+/-) | (+/-) | 73 | 4.01 | Married | South central | II |
| P316 | (+/+) | (-/-) | (+/+) | 71 | 3.04 | Widow | South central | II |
| P317 | (+/+) | (+/+) | (-/-) | 66 | 2.02 | Married | South central | I |
| P318 | (+/+) | (-/-) | (+/+) | NA | NA | Divorced | NA | NA |
| **Table S2. (Cont.)** | | | |  |  |  |  |  |
|  |  |  |  |  |  |  |  |  |
| ID | *CYP3A4*1B* Genotype | *PXR-HNF3beta* Genotype | *PXR-HNF4* Genotype | Age | PSA | Marital status | Residence area | DRE |
| P319 | (+/+) | (+/-) | (+/-) | 68 | 2.14 | Married | South central | I |
| P320 | NA | NA | NA | NA | NA | NA | NA | NA |
| P321 | (+/+) | (+/+) | (-/-) | 66 | 1.14 | Married | South central | II |
| P322 | (+/+) | (+/+) | (-/-) | 61 | 2.51 | Married | South central | II |
| P323 | (+/+) | (+/+) | (+/-) | 80 | 2.26 | Single | South central | II |
| P324 | (+/-) | (+/+) | (-/-) | 63 | 1.53 | Married | South central | II |
| P325 | (+/+) | (+/-) | (+/-) | 69 | 3.37 | Married | South central | I |
| P326 | (+/+) | (+/-) | (+/-) | 60 | 0.97 | Cohabitation | South central | I |
| P327 | (+/-) | (+/-) | (+/-) | 70 | 0.3 | Single | South central | II |
| P328 | (+/+) | (+/-) | (+/-) | 70 | 0.135 | Single | East | I |
| P329 | NA | NA | NA | NA | NA | NA | NA | NA |
| P330 | (+/+) | (-/-) | (+/+) | 70 | 2.53 | Married | South central | I |
| P331 | (+/+) | (+/+) | (-/-) | 63 | 2.07 | Married | South central | II |
| P332 | (+/+) | (+/+) | (-/-) | 87 | 3.5 | Married | South central | II |
| P333 | (+/+) | (+/-) | (+/-) | 77 | 2.05 | Single | South central | II |
| P334 | (+/+) | (+/-) | (-/-) | NA | NA | NA | NA | NA |
| P335 | NA | NA | NA | 63 | 1.82 | NA | South central | II |
| P336 | (+/-) | (+/+) | (-/-) | 65 | 1.64 | Married | South central | I |
| P337 | (+/+) | (+/+) | (-/-) | 63 | 1.38 | Married | South central | III |
| P338 | (+/+) | (+/-) | (+/+) | 61 | 2.67 | Married | South central | I |
| P339 | (+/-) | (+/-) | (+/-) | 64 | 0.27 | Married | South central | II |
| P340 | (+/+) | (+/+) | (-/-) | 63 | 1.67 | Married | South central | I |
| P341 | (+/+) | (+/-) | (-/-) | 62 | 1.79 | Married | South central | NA |
| P342 | (+/-) | (+/-) | (+/-) | 67 | 2.02 | Married | South central | II |
| P343 | (+/+) | (+/+) | (+/-) | 62 | 0.9 | Divorced | South central | I |
| P344 | (+/+) | (+/+) | (-/-) | 62 | 0.27 | Divorced | South central | NA |
| P345 | (+/+) | (-/-) | (+/+) | 75 | 2.67 | Married | West | I |
| P346 | (+/-) | (+/+) | (+/+) | 61 | 2.37 | Single | South central | II |
| P347 | (+/+) | (+/+) | (+/-) | 63 | 2 | Married | South central | II |

(+/+) = Wild-type variant, homozygous.

(+/-) = Heterozygous.

(-/-) = Polymorphic variant, homozygous.

NA, low DNA quality to perform analysis and/or missing data from medical records.
