# Supplementary material for: The PXR rs7643645 Polymorphism Is Associated with the Risk of Higher Prostate-Specific Antigen Levels in Prostate Cancer Patients
Source: PLoS One. 2014 Jun 12;9(6):e99974. doi: 10.1371/journal.pone.0099974 (PMC4055777; doi:10.1371/journal.pone.0099974)
Supplement: Table S3 — Relation between CYP3A4 and PXR genotypes and clinical characteristics among cases. (DOC) [file pone.0099974.s003.doc]

| **Table S3. Relation between *CYP3A4* and *PXR* genotypes and clinical characteristics among cases.** | | | | | |
| --- | --- | --- | --- | --- | --- |
|  | Genotype |  |  | OR (95% CI) | ^*^p value |
| PSA  (*n*=93) |  | PSA ≤ 10 | PSA > 10 |  |  |
|  | *CYP3A4* | n (%) | n (%) |  |  |
|  | **1A/*1A* | 25 (83.33) | 52 (82.54) | 1 |  |
|  | **1A/*1B* | 5 (16.66) | 7 (11.11) | 0.61 (0.17-2.11) | 0.43 |
|  | **1B/*1B* | 0 (0.00) | 4 (6.35) | NA | NA |
|  | *PXR-HNF3β* |  |  |  |  |
|  | *WT/WT* | 10 (33.33) | 25 (39.68) | 1 |  |
|  | *WT/T* | 14 (46.66) | 30 (47.61) | 1.80 (0.50-6.36) | 0.36 |
|  | *T/T* | 6 (20.00) | 8 (12.69) | 1.53 (0.45-5.21) | 0.48 |
|  | *PXR-HNF4* |  |  |  |  |
|  | *WT/WT* | 10 (33.33) | 10 (15.87) | 1 |  |
|  | *WT/G* | 14 (46.66) | 31 (49.20) | 2.46 (0.82-7.30) | 0.10 |
|  | *G/G* | 6 (20.00) | 22 (34.92) | 3.99 (1.14-13.99) | **0.03** |
| Gleason grade  (*n*=86) | | Gleason score ≤ 7 | Gleason score > 7 |  |  |
|  | *CYP3A4* | n (%) | n (%) |  |  |
|  | **1A/*1A* | 33 (76.74) | 38 (88.37) | 1 |  |
|  | **1A/*1B* | 7 (16.28) | 4 (9.30) | 0.51 (0.13-1.90) | 0.31 |
|  | **1B/*1B* | 3 (6.98) | 1 (2.33) | 0.29 (0.02-3.00) | 0.30 |
|  | *PXR-HNF3β* |  |  |  |  |
|  | *WT/WT* | 16 (37.21) | 16 (37.21) | 1 |  |
|  | *WT/T* | 20 (46.51) | 20 (46.51) | 0.89 (0.35-2.26) | 0.81 |
|  | *T/T* | 7 (16.28) | 7 (16.28) | 0.82 (0.24-2.79) | 0.75 |
|  | *PXR-HNF4* |  |  |  |  |
|  | *WT/WT* | 9 (20.93) | 10 (23.26) | 1 |  |
|  | *WT/G* | 21 (48.84) | 20 (46.51) | 0.85 (0.28-2.54) | 0.78 |
|  | *G/G* | 13 (30.23) | 13 (30.23) | 0.83 (0.25-2.70) | 0.76 |
| TNM score  (*n*=83) |  | TNM ≤ 2 | TNM ≥ 3 |  |  |
|  | *CYP3A4* | n (%) | n (%) |  |  |
|  | **1A/*1A* | 57 (85.07) | 12 (75.0) | 1 |  |
|  | **1A/*1B* | 6 (8.96) | 4 (25.0) | 3.16 (0.77-12.97) | 0.10 |
|  | **1B/*1B* | 4 (5.97) | 0 (0.00) | NA | NA |
|  | *PXR-HNF3β* |  |  |  |  |
|  | *WT/WT* | 26 (38.81) | 6 (37.5) | 1 |  |
|  | *WT/T* | 29 (43.28) | 8 (50.0) | 1.28 (0.39-4.19) | 0.67 |
|  | *T/T* | 12 (17.91) | 2 (12.5) | 0.75 (0.13-4.26) | 0.74 |
|  | *PXR-HNF4* |  |  |  |  |
|  | *WT/WT* | 16 (23.88) | 2 (12.5) | 1 |  |
|  | *WT/G* | 27 (40.30) | 10 (62.5) | 2.96 (0.57-15.26) | 0.19 |
|  | *G/G* | 24 (35.82) | 4 (25.0) | 1.33 (0.21-8.15) | 0.75 |
|  |  |  |  |  |  |

PSA, prostate-specific antigen. TNM, tumor lymph nodes metastasis. NA, not applicable. ^*^Logistic model.

n, No. of subjects.
