# Supplementary material for: The PXR rs7643645 Polymorphism Is Associated with the Risk of Higher Prostate-Specific Antigen Levels in Prostate Cancer Patients
Source: PLoS One. 2014 Jun 12;9(6):e99974. doi: 10.1371/journal.pone.0099974 (PMC4055777; doi:10.1371/journal.pone.0099974)
Supplement: Table S4 — CYP3A4 and PXR allelic frequencies. (DOC) [file pone.0099974.s004.doc]

**Table S4. *CYP3A4* and *PXR* allelic frequenies**

|  | number (%) | | Frequency | | *p value |
| --- | --- | --- | --- | --- | --- |
| *CYP3A4* | Cases | Controls | Cases | Controls |  |
| **1A* | 178 (89.9) | 260 (90.3) | 0.10 | 0.10 | 0.8906 |
| **1B* | 20 (10.1) | 28 (9.7) |
| *PXR-HNF3β* |  |  |  |  |  |
| WT | 123 (62.12) | 172 (59.7) | 0.38 | 0.40 | 0.5947 |
| T | 75 (37.88) | 116 (40.3) |
| *PXR-HNF4* |  |  |  |  |  |
| WT | 87 (43.9) | 127 (44.1) | 0.56 | 0.56 | 0. 9725 |
| G | 111 (56.1) | 161 (55.9) |

*X^2^ test.
